# Carotid Body Tumor: A Case Report of a Rare Neuroendocrine Neoplasm

**DOI:** 10.7759/cureus.42224

**Published:** 2023-07-20

**Authors:** Ali J Alqhtani, Samar M Algahtani, Mohammed K Alharbi, Muaath H Aljehani, Ahlam Alharbi

**Affiliations:** 1 Radiology, King Abdulaziz Hospital, Jeddah, SAU; 2 Collage of Medicine, King Saud Bin Abdulaziz University for Health Sciences, Riyadh, SAU; 3 General Practice, Al-Rayan Colleges, Madinah, SAU; 4 General Practice, King Saud University, Riyadh, SAU; 5 Family Medicine, Primary Health Care Center, Riyadh, SAU

**Keywords:** case report, paraganglioma, computed tomography, carotid body tumor, neck mass

## Abstract

Carotid body tumors are rare neuroendocrine tumors originating from paraganglionic cells in the carotid body. Although these tumors are typically slow-growing and benign, their location and vascularity present unique challenges in management. Here, we present a case study of a 58-year-old male who presented with a painless, gradually enlarging neck mass over a six-month period. Physical examination revealed a non-tender, pulsatile mass measuring approximately 3 cm in the left carotid triangle. Imaging studies, including ultrasound and contrast-enhanced computed tomography, confirmed the presence of a well-defined, hypoechoic mass at the left carotid bifurcation, displacing adjacent vessels. A multidisciplinary team planned surgical resection, guided by imaging, resulting in the successful removal of the tumor. Histopathological examination confirmed the diagnosis of a carotid body tumor. This case report underscores the significance of accurate diagnosis, a multidisciplinary approach, and advanced imaging techniques in managing carotid body tumors. Surgical resection, guided by imaging, aims to achieve complete excision while preserving vital structures. Long-term follow-up is crucial to detect potential recurrence or progression early.

## Introduction

Carotid body tumors, also known as carotid body paragangliomas, are rare neuroendocrine tumors that originate from the paraganglionic cells of the carotid body. The carotid body is a small chemoreceptor located at the bifurcation of the common carotid artery. These tumors represent a small percentage of neoplasms in the head and neck region, accounting for approximately 0.5% of cases [1]. Carotid body tumors are typically characterized by slow growth and have a predominantly benign nature, but their location and vascularity can present challenges in their management. The clinical presentation of carotid body tumors often involves the identification of a painless and slowly growing mass in the neck. Although many tumors remain asymptomatic, larger tumors can cause symptoms related to local compressions, such as dysphagia, hoarseness, or respiratory difficulties. Accurate diagnosis relies on imaging modalities to assess the extent of the tumor and aid in surgical planning [2].

## Case presentation

A 58-year-old male presented with a painless neck mass that had gradually increased in size over the past six months. The patient reported no associated symptoms, such as difficulty breathing, swallowing, or changes in voice. His medical history included well-controlled hypertension, and there were no known allergies. There was no family history of similar neck masses or relevant genetic conditions. The patient did not smoke and did not consume alcohol regularly.

The patient first noticed a small, painless lump in his neck six months ago. Over time, the lump gradually increased in size without associated symptoms. Concerned about the enlarging neck mass, the patient sought medical evaluation.

On physical examination, a non-tender, firm, mobile mass measuring approximately 3 cm in diameter was palpable in the left carotid triangle. The mass exhibited a pulsatile nature and was located anterior to the sternocleidomastoid muscle. No other significant physical findings were observed, and the patient's vital signs were within normal limits.

**Figure 1:**
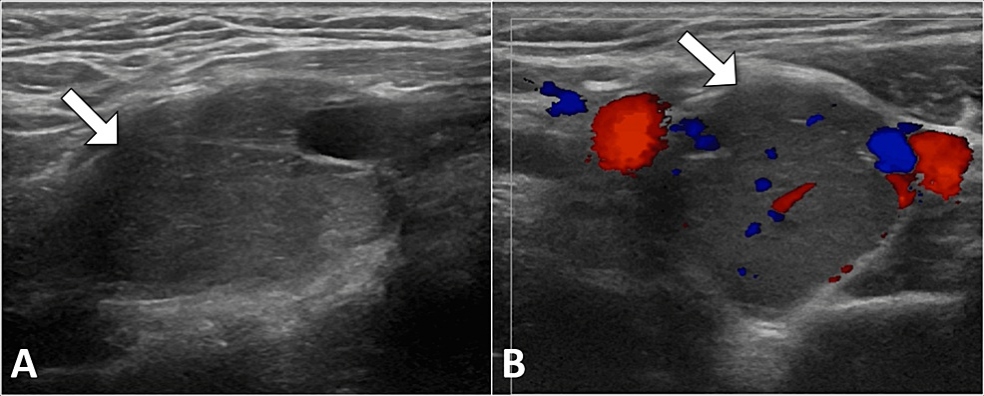
Longitudinal grayscale (A) and transverse color Doppler views of the neck displaying a solid mass (arrow) causing displacement of the internal and external carotid arteries without invasion.

Ultrasound imaging of the neck was performed, revealing a well-defined, hypoechoic mass located at the left carotid bifurcation. Doppler examination demonstrated a solid vascular mass that displaced the internal and external carotid arteries without direct invasion. No suspicious lymphadenopathy or other abnormal findings were detected (Figure 1).

**Figure 2:**
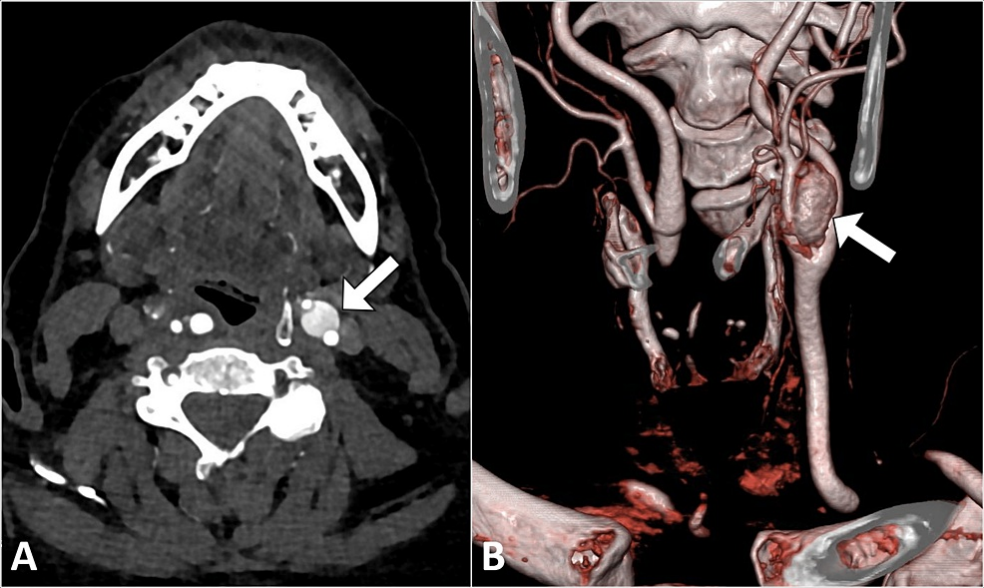
Axial contrast-enhanced CT image (A) of the neck revealing a prominently enhancing mass (arrow) located in the notch between the internal and external carotid arteries. Three-dimensional volume-rendered CT image (B) illustrating the carotid body tumor (arrow).

To further evaluate the characteristics and extent of the mass, a contrast-enhanced computed tomography (CT) scan of the neck was conducted. The CT scan confirmed the presence of a well-defined, enhancing soft tissue mass measuring 3.2 cm x 2.8 cm in the left carotid bifurcation region. The mass displaced adjacent vessels, with the left internal carotid artery being displaced posterolaterally and the left external carotid artery being displaced anteromedially. The internal jugular vein was visualized posterior to the mass (Figure 2).

Given the size and location of the tumor, surgical resection was planned as the primary therapeutic intervention. A multidisciplinary team, including vascular surgeons and head and neck surgeons, was involved in developing the surgical plan. Preoperative embolization was considered to reduce the risk of intraoperative bleeding. Under general anesthesia, meticulous dissection and excision of the tumor were performed while preserving the integrity of the carotid artery and other vital structures.

**Figure 3:**
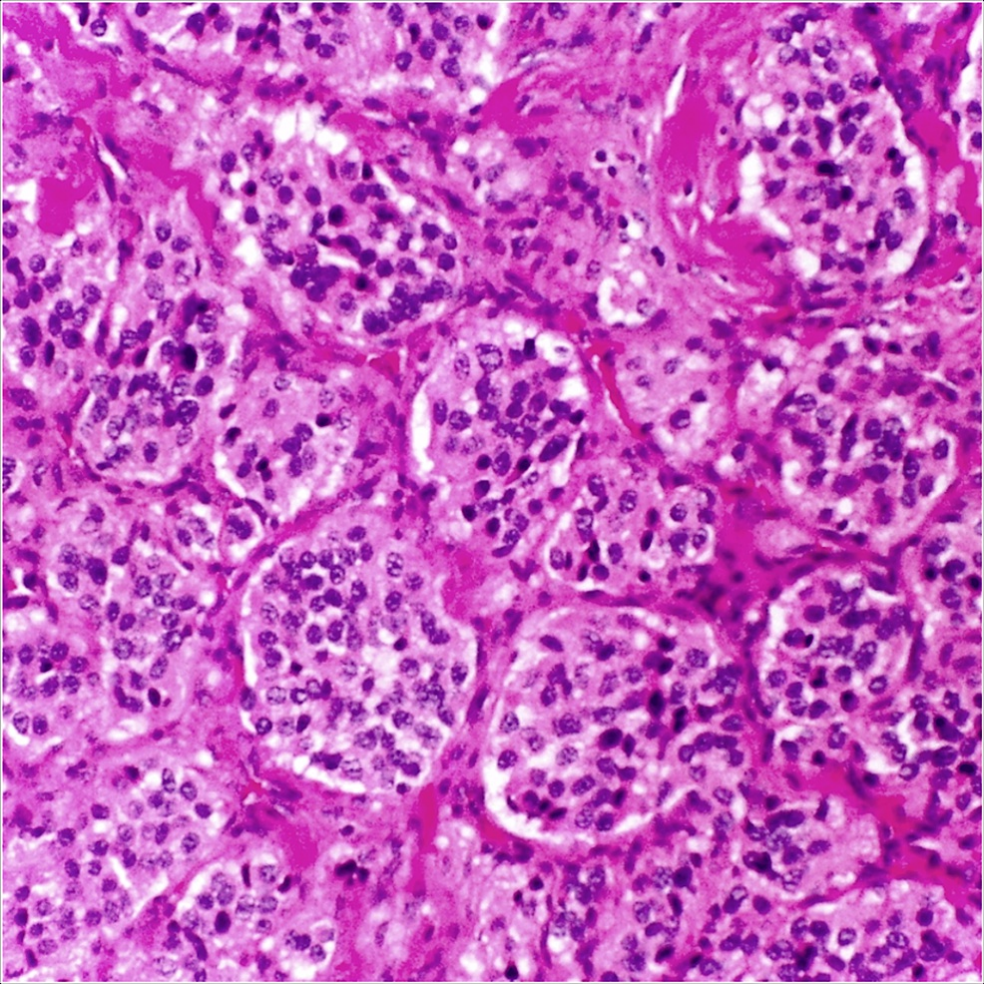
Histopathology image demonstrating the characteristic pattern of epithelioid cells arranged in distinct clusters, interspersed with a prominent capillary network, consistent with a carotid body tumor.

Histopathological examination of the excised tumor revealed the prevailing histologic pattern of epithelioid cells arranged in distinct clusters separated by a prominent capillary network. These findings were consistent with the diagnosis of a carotid body tumor (Figure 3). The patient underwent regular follow-up appointments to monitor for any signs of recurrence and assess postoperative recovery.

## Discussion

Carotid body tumors are rare neuroendocrine tumors that originate from the paraganglionic cells of the carotid body. Accurate preoperative diagnosis of carotid body tumors is essential for appropriate management and surgical planning [3]. In our case report, we utilized multiple imaging modalities to confirm the diagnosis and assess tumor characteristics.

Doppler ultrasound is a valuable initial imaging modality for carotid body tumors, offering high specificity and sensitivity. It enables the identification of a well-defined, hypoechoic mass located at the carotid bifurcation, often accompanied by a hypervascular appearance and low-resistance arterial flow pattern [3,4].

Further imaging evaluation with contrast-enhanced CT provides additional details regarding tumor extent and its relationship with adjacent structures. In our case, CT imaging demonstrated a homogeneously enhancing soft tissue mass, exhibiting the characteristic splaying of the carotid vessels. This "Goblet deformity" appearance aids in distinguishing carotid body tumors from other neck masses and guides surgical planning. However, it is important to consider the limitations of CT, such as the use of contrast media and ionizing radiation, especially in long-term follow-up [5].

MRI is considered the criterion standard for assessing carotid body tumors due to its excellent soft tissue visualization and lack of ionizing radiation. MRI findings typically show hyperintense lesions with a "salt and pepper" appearance, representing high-flow vascular voids within the tumor. This detailed imaging allows for the accurate evaluation of tumor characteristics, including size, location, and involvement of nearby structures [6].

While imaging plays a crucial role in the diagnosis and preoperative evaluation of carotid body tumors, treatment decisions should be based on a multidisciplinary approach. Surgical resection remains the primary treatment modality for carotid body tumors, aiming for complete excision while preserving the integrity of vital structures, such as the carotid artery and adjacent nerves. However, the use of radiotherapy as a primary treatment approach for carotid body tumors has been debated. Some studies suggest that these tumors are not highly radiosensitive and may exhibit regrowth after suppression. Therefore, surgery is usually preferred for younger, healthier patients, while radiotherapy is considered for elderly patients or individuals who are poor surgical candidates [5,6].

## Conclusions

This case report highlights the diagnostic evaluation, treatment approach, and follow-up of a patient with a carotid body tumor. The utilization of imaging modalities played a crucial role in the accurate diagnosis and preoperative assessment of the tumor. Surgical resection remains the primary treatment modality for carotid body tumors, aiming for complete excision while preserving vital structures. Long-term follow-up is necessary to monitor for the recurrence or progression of the tumor. This case emphasizes the importance of a multidisciplinary approach, adherence to established guidelines, and close collaboration among healthcare professionals to optimize patient outcomes in the management of carotid body tumors.
